# Supplementary material for: On the Road Safety: Gender Differences in Risk-Taking Driving Behaviors Among Seniors Aged 65 and Older
Source: Geriatrics (Basel). 2024 Oct 21;9(5):136. doi: 10.3390/geriatrics9050136 (PMC11507166; doi:10.3390/geriatrics9050136)
Supplement: Supplementary file 1 [file geriatrics-09-00136-s001.zip › Supplementary Material A.pdf]

Driver Road Risk Perception Scale (DRPS)

Read each statement and indicate how risky you perceive the described situation to be, choosing the most appropriate response for each statement.

	Not at all risky			Extremely risky
1. Eating while driving (a simple sandwich, snack, etc.):	1	2	3	4	5
2. Reading a text message on the phone while driving:	1	2	3	4	5
3. Typing a message on the phone while driving:	1	2	3	4	5
4. Driving the car after an evening of drinking a few glasses of wine, beer, or alcoholic cocktails:	1	2	3	4	5
5. Putting yourself behind the wheel after taking medications without checking for possible interactions:	1	2	3	4	5
6. Talking on the phone without an earpiece while driving:	1	2	3	4	5
7. Consulting and/or typing while driving to look up the road with the navigator:	1	2	3	4	5
8. Putting oneself at the wheel under conditions of particular physical fatigue:	1	2	3	4	5
9. Using the phone while driving to take photos (traffic situation, landscape around, etc.):	1	2	3	4	5
10. Arguing animatedly with a passenger while driving:	1	2	3	4	5

11. Not wearing seat belts while sitting in the front seats of the vehicle:	1	2	3	4	5
12. Not wearing seat belts when sitting in the rear seats of the vehicle:	1	2	3	4	5
13. Carrying a child in the car without securing the child in the appropriate seat:	1	2	3	4	5
