# Supplementary material for: On the Road Safety: Gender Differences in Risk-Taking Driving Behaviors Among Seniors Aged 65 and Older
Source: Geriatrics (Basel). 2024 Oct 21;9(5):136. doi: 10.3390/geriatrics9050136 (PMC11507166; doi:10.3390/geriatrics9050136)
Supplement: Supplementary file 1 [file geriatrics-09-00136-s001.zip › Supplementary Materials B.pdf]

Pedestrian Behavior Appropriateness Perception Scale (PBAPS)

How appropriate do you think the following pedestrian behaviors are:

	Not at all				Somewhat					Completely
1. While waiting for a grandchild to come out of School, occupy the roadside if the sidewalk is full of people:	1	2	3	4	5	6	7	8	9	10
2. Read a text message on the phone while crossing the street if the road has little traffic:	1	2	3	4	5	6	7	8	9	10
3. Chatting with friends on a street (off the sidewalk) in the center of one's city or town, since cars have to go very slowly in urban centers:	1	2	3	4	5	6	7	8	9	10
4. Crossing where there is no crosswalk if cars are still far away:	1	2	3	4	5	6	7	8	9	10
5. Cross when the pedestrian light is red if you are in a hurry:	1	2	3	4	5	6	7	8	9	10
6. Cross the road while a vehicle is coming over at moderate speed, at a distance of 20 meters from you:	1	2	3	4	5	6	7	8	9	10
7. Cross the street in front of the stopped bus to allow travelers to get off:	1	2	3	4	5	6	7	8	9	10
